# Small bowel obstruction due to a congenital adhesion: a rare case report

**DOI:** 10.1093/jscr/rjab282

**Published:** 2021-07-05

**Authors:** Kostas Tepelenis, Stefanos K Stefanou, Christos K Stefanou, Nikolaos Tepelenis, Persefoni Margariti, Amalia Christopoulou, George Gogos-Pappas, Konstantinos Vlachos

**Affiliations:** Department of Surgery, University Hospital of Ioannina, Ioannina, Greece; Department of Surgery, General Hospital of Ioannina G. Xatzikosta, Ioannina, Greece; Department of Surgery, General Hospital of Filiates, Filiates, Greece; Department of Pathology, Agia Sofia Children’s Hospital, Athens, Greece; Department of Radiology, University Hospital of Ioannina, Ioannina, Greece; Department of Anesthesiology, University Hospital of Ioannina, Ioannina, Greece; Department of Surgery, University Hospital of Ioannina, Ioannina, Greece; Department of Surgery, University Hospital of Ioannina, Ioannina, Greece

**Keywords:** adhesions, post-surgical adhesions, post-inflammatory adhesions, congenital adhesions, small bowel obstruction

## Abstract

The exact incidence of small bowel obstruction (SBO) due to congenital adhesions remains unclear. Herein, we report a 59-year-old male who appeared in the emergency department with diffuse abdominal pain associated with vomiting. The patient reported no previous medical or surgical history. Clinical examination revealed a soft, distended abdomen and diffuse tenderness. Computed tomography indicated a close loop obstruction. A congenital band extending from mesentery to ileum and causing an internal hernia was identified via a midline incision. The band was ligated and divided. There is no difference in the clinical presentation, and the initial work-up of SBO on account of congenital adhesions was compared to other bowel obstruction causes. Surgical exploration is crucial for the diagnosis and treatment of congenital adhesions. Although laparotomy is considered the cornerstone of surgical management, laparoscopy has emerged as a feasible and safe alternative for the diagnosis and treatment of these congenital bands.

## INTRODUCTION

Congenital adhesions are infrequent in children and is rare in adults. Ileum is the commonest site of congenital bands, followed by colon, mesentery, omentum, peritoneum, jejunum as well as every site of the gastrointestinal tract, including the abdominal organs and peritoneum [1].

There are no differences in the clinical manifestation and initial work-up of small bowel obstruction (SBO) due to congenital bands when compared to other causes of SBO [2]. Physical examination and imaging modalities, particularly computed tomography (CT) scan, are helpful to exclude other causes of SBO. Surgical exploration is essential for the diagnosis and treatment of congenital adhesions. In recent years, a laparoscopic approach is considered as a feasible, safe and low-risk option for the management of adhesive SBO [3].

To the best of our knowledge, 57 cases of SBO due to congenital bands in adults have been reported in the literature until now. Here, we report the case of a 59-year-old male who was diagnosed with SBO due to a congenital adhesion.

## CASE REPORT

A 59-year-old-male visited the emergency department with a history of diffuse abdominal pain in the previous 6 h. The pain was described as colicky, and it was associated with vomiting. The patient reported no previous medical or surgical history. Abdominal examination revealed a soft, distended abdomen, tympanic on percussion, diffuse tenderness on palpation and positive bowel sounds. No rebound pain or muscle guarding were revealed by the physical examination.

**Figure 1:**
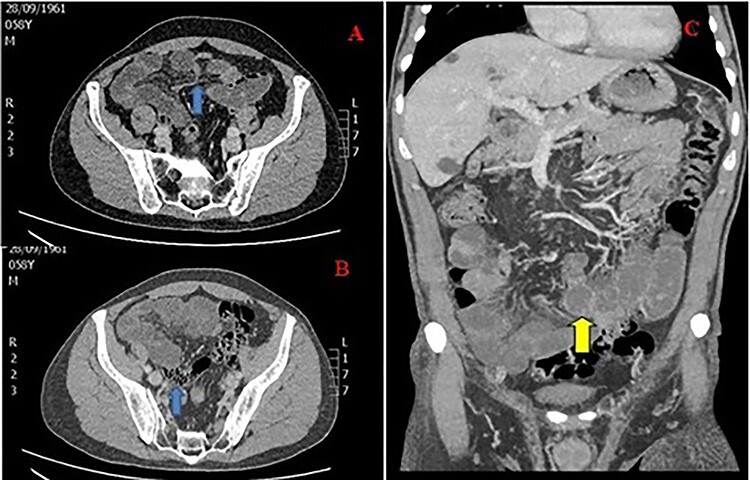
CT of the abdomen in a 59-year-old male with SBO; (**A**) transition point in the right iliac fossa (arrow), (**B**) feces sign (arrow) and (**C**) close loop obstruction (arrow).

Vital signs were as follows: blood pressure (BP): 163/93 mmHg, heart rate (HR): 80/min, respiratory rate (RR): 14/min, SO2: 99% and temperature (T): 36.5°C. Laboratory studies were unremarkable. A contrast-enhanced CT of the abdomen was performed, and it showed distended jejunal and ileum loops with a transition point in the right iliac fossa after which ileal loops, as well as the large bowel, were collapsed. The small bowel feces sign was also observed. The CT of the abdomen corroborated the diagnosis of SBO and indicated a close loop obstruction (Fig. 1).

The patient was then transferred to the operating room for exploratory laparotomy via a midline incision. A congenital band obstructing the ileum was observed. This anomalous band extending from mesentery to ileum caused a small window through which internal herniation of the small bowel and obstruction occurred. No signs of bowel ischemia or peritonitis were noted. The band was ligated and divided. The patient recovered uneventfully, and he was discharged after 3 days.

## DISCUSSION

Adhesive SBO is a frequent clinical problem worldwide that results in hospitalization and, quite often, in surgical exploration [4]. Touloukian first described this entity in 1979 [5]. Adhesions are usually developed after surgical operations (especially colorectal, oncologic gynecological or pediatric surgery), abdominal trauma and inflammation [4].

Despite being infrequently in children [5] and rare in adults [6], congenital bands can pose SBO at any age. The exact incidence of SBO due to these bands remains unclear [2]. The ileum is the most typical location of congenital bands. Other sites encompass the colon, mesentery, omentum, peritoneum, jejunum and every site of the gastrointestinal tract, including abdominal organs and peritoneum [1, 5–7].

Adhesive SBO manifests the clinical symptoms and signs of SBO regardless of the etiology. Occlusion occurs either from the small bowel’s compression due to these bands or trapping of the small bowel loops between the band and the mesentery [2]. The clinical presentation includes colicky abdominal pain, nausea and distention with or without vomiting and obstipation. Physical examination is crucial for recognizing the signs of strangulation and exclusion of any abdominal wall or groin hernias [3]. Indications of strangulation encompass steady, severe pain, tenderness and minimal peristalsis or silent abdomen in the auscultation [1].

Laboratory studies that might indicate peritonitis include elevated white blood cell count and c-reactive protein, though these tests’ sensitivity and specificity are relatively low. Plain X-rays are non-specific, with a sensitivity of 70% in the diagnosis of SBO. Typical features encompass the distention of small bowel loops, multiple air-fluid levels and gas absence in the colon. Plain X-rays can also detect pneumoperitoneum secondary to bowel perforation [8, 9]. CT scan diagnoses SBO with high accuracy, detects obstruction location, differentiates accurately between different causes of SBO by excluding other causes and displays a 90% accuracy in predicting strangulation and the need for urgent surgery. Signs indicating strangulation include close loop, bowel ischemia and free fluid in the abdomen [3, 8, 10]. Water-soluble contrast studies predict the need for surgery. Suppose the contrast does not reach the colon on an abdominal X-ray taken 24 h after the contrast administration. In that case, non-operative management seems to fail, and surgery is recommended. These studies also decrease the need for surgery due to the contrast’s active therapeutic role [11, 12].

Surgical exploration is the cornerstone of the management of congenital adhesions causing SBO as it is the only way to establish the final diagnosis and treatment [2]. In recent years, laparoscopic exploration has gained much popularity and is considered a safe and low-risk option for diagnosing and treating adhesive SBO [3]. The potential benefits of laparoscopy are the shorter length of stay, reduced operative time, lower postoperative pain, earlier return of bowel movements, less extensive adhesion reformation, decreased 30-day mortality, lower morbidity and fewer major postoperative complications [4, 13, 14]. On the contrary, the estimated bowel injury with laparoscopic adhesiolysis is 6.3–26.9% of cases. Additionally, bowel resection was more frequent in laparoscopic surgery than laparotomy (53.5 vs. 43.4%) [3].

Inclusion criteria for laparoscopic approach encompass mild abdominal distention, a proximal obstruction, a partial obstruction and an anticipated single-band obstruction [7]. The predictive factors for successful laparoscopic adhesiolysis include less than or equal to two previous laparotomies, appendectomy as the operation in history, no previous median laparotomy, single adhesive band, no signs of peritonitis, early laparoscopic management within 24 h from the onset of symptoms and experience of the surgeon. Conversion to laparotomy is not a failure but a wise option in certain circumstances. The conversion rate varies between 0 and 52%, depending on the patient selection and surgical skill [2, 15].
